# Electrocoagulation-free strategy in minimally invasive direct coronary artery bypass with hybrid revascularisation – a case report

**DOI:** 10.1186/s13019-024-03203-x

**Published:** 2025-01-20

**Authors:** Carla L. Schuering, Leonhard Wert, Johanna K. R. von Mackensen, Vanessa I. T. Zwaans, Julius Kaemmel, Roland Heck, Christoph T. Starck, Jörg Kempfert, Stephan Jacobs, Volkmar Falk, Alaa Abd El Al

**Affiliations:** 1 Department of Cardiothoracic and Vascular Surgery, Deutsches Herzzentrum der Charité (DHZC), Augustenburger Platz 1, 13353 Berlin, Germany; 2 DZHK (German Center for Cardiovascular Research), Partner Site Berlin, Berlin, Germany; 3 Department of Cardiothoracic Surgery, Charité − Universitätsmedizin Berlin, Corporate Member of Freie Universität Berlin, Humboldt-Universität zu Berlin, Charitéplatz 1, 10117 Berlin, Germany; 4 Department of Health Sciences and Technology, ETH Zurich, Zurich, Switzerland

## Abstract

**Background:**

Hybrid coronary revascularisation benefits patients with multivessel disease, as it amalgamates the minimally invasive direct coronary artery bypass (MIDCAB) procedure and percutaneous coronary intervention (PCI).

**Case summary:**

We present a 63-year-old female with triple-vessel coronary artery disease including marked ostial stenosis of the left main coronary artery, as well as moderate stenosis of the right coronary artery. The risk of death following heart surgery (EuroSCORE II) is 4.27%. The patient exhibited multiple morbidities including chronic obstructive pulmonary disease, renal impairment, extracardiac arteriopathy, and multiple prior gastrointestinal surgeries, as well as a recent episode of paroxysmal atrial fibrillation. A MIDCAB procedure without electrocoagulation was stipulated by the ENT specialist due to the patient’s cochlear implant.

**Conclusion:**

A successful MIDCAB procedure omitting electrocoagulation was performed for the first time for multivessel coronary disease in a multimorbid patient as part of a hybrid approach.

**Supplementary Information:**

The online version contains supplementary material available at 10.1186/s13019-024-03203-x.

## Introduction

Since its first introduction by Calafiore et al. in 1996, minimally invasive direct coronary artery bypass (MIDCAB) grafting has become the routine procedure in patients with isolated proximal stenosis of the left anterior descending coronary artery (LAD). Initially designed as a less invasive, sternal-sparing approach to conventional coronary artery bypass grafting (CABG), it is meant to enable revascularisation of the LAD with the left internal mammary artery (LIMA) via a small left anterior thoracotomy [1]. Today, this surgical technique has evolved and has also proven successful in selected cases of multivessel coronary artery disease (MVCAD) as part of a MIDCAB + PCI hybrid approach [2]. The reduced surgical trauma associated with circumventing sternotomy and avoiding cardiopulmonary bypass (CPB) has resulted in fewer bleeding events, fewer transfusions, faster recoveries and a reduced risk of wound infections [3].

## Case presentation

### Patient details

In June 2023, a 63-year-old female patient (77 kg, 158 cm, BMI 30.8 kg/m^2^, BSA 1.79 m^2^) presented with dyspnoea on exertion consistent with NYHA class III. The patient’s recent history included infection-triggered paroxysmal atrial fibrillation which spontaneously reverted to sinus rhythm under inpatient beta-blocker treatment. At the time, the patient was started on direct oral anticoagulation. Additionally, the patient presented with hyperlipidaemia and essential arterial hypertension under quadruple antihypertensive treatment. Extracardiac arteriopathies included right carotid stenosis, which was treated surgically in 2018.

The patient presented with a history of nicotine abuse and moderate chronic obstructive pulmonary disease (COPD), consistent with GOLD class 2, as well as stage 4 chronic kidney disease, all in line with American Society of Anesthesiology (ASA) class III of severe systemic disease. The patient underwent multiple gastric and intestinal surgeries, including a Billroth II procedure in 1986 with reoperation in 2020, a hysterectomy in 1989, and a salpingo-oophorectomy in 2014. The patient was being treated for depression and for hypothyroidism with Hashimoto’s disease at the time of admission.

Since a sepsis episode in 2014, the patient developed gait insecurity and inner ear damage secondary to ciprofloxacin administration. The patient received a left hearing aid and a right cochlear implant. Positive preoperative screening for methicillin-resistant Staphylococcus aureus (MRSA) confirmed the need for appropriate isolation. Elevated infectious disease parameters and a suspected bronchopulmonary infection prompted the initiation of a standardised 5-day MRSA eradication regimen.

The right coronary artery (RCA) showed moderate stenosis, which is hemodynamically significant according to preoperative fractional flow reserve measurement (value ≤ 0.80) [4]. Marked ostial narrowing was found in the main trunk of the left coronary artery (LCA). Given its intramural position and thin calibre, the LAD was identified as atypical. Its first diagonal branch (D1) was established as a long vessel running over the anterior wall with a strongly tortuous course around the heart’s apex, with no signs of narrowing. The circumflex branch (RCX) showed a small supply area and no relevant stenosis.

Preoperative transthoracic echocardiography showed a preserved left ventricular systolic function of 0.58 using Simpson’s biplane method of discs, as well as normal valvular function. A small pleural effusion was identified on the left side.

Considering these findings, our heart team recommended an electrocoagulation-free approach including hybrid coronary revascularisation (HCR) with LIMA-to-LAD bypass surgery via MIDCAB followed by future interventional stenting of the RCA. This is consistent with the preoperative ENT consultation which discouraged the use of monoterminal electrosurgery due to the potential direct coupling between the cochlear implant electrode array and the cautery tip [5]. Even though the use of bipolar electrocoagulation at a distance of at least 4 cm from the cochlear implant is approved, and supported by the British Cochlear Implant Group (BCIG) guidelines [6], our aim was to avoid using it due to the paucity of studies regarding proper instrument use in patients with cochlear implants and the ambiguity of current recommendations [7].

### Surgical procedure

**Fig. 1:**
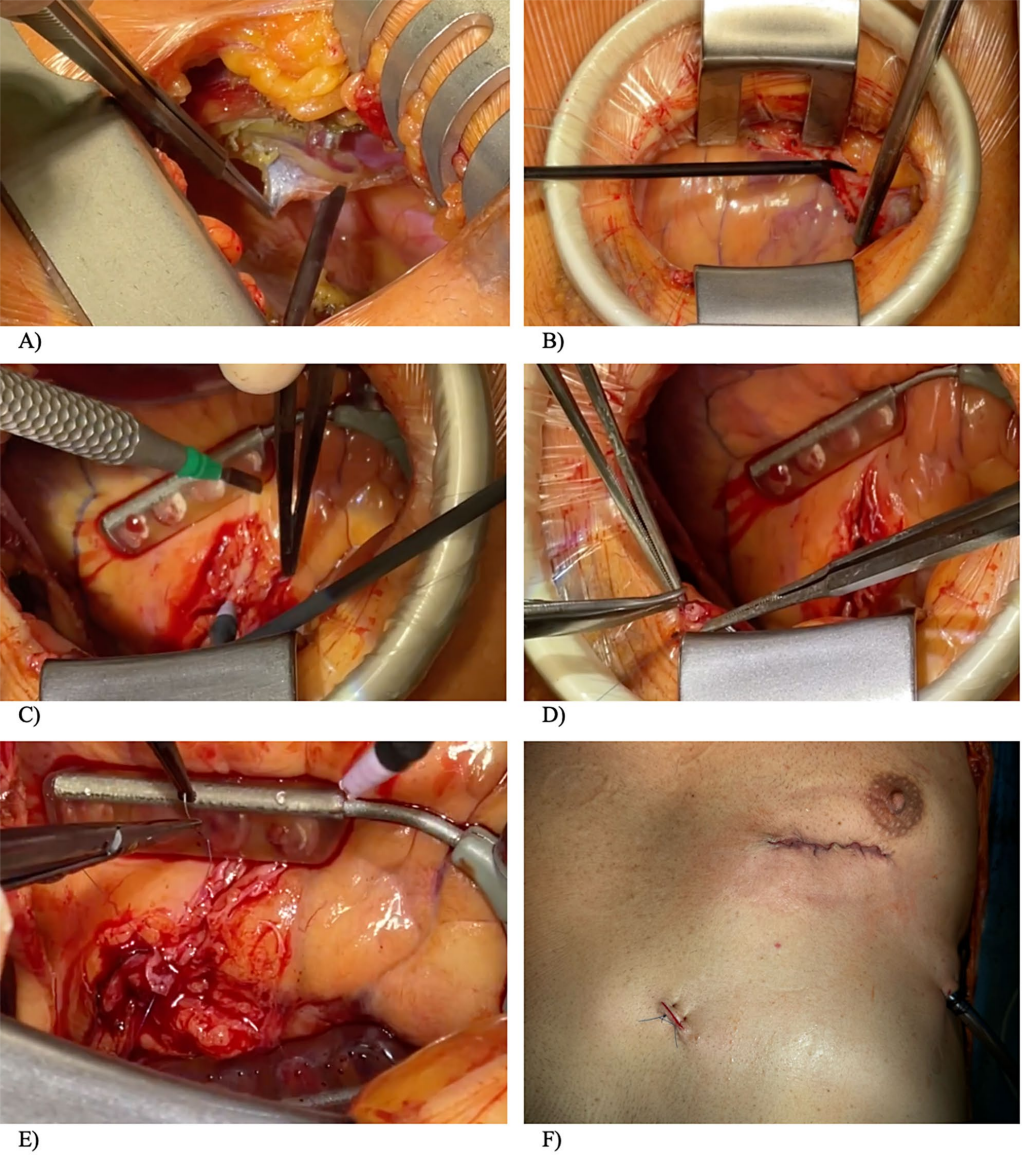
MIDCAB Procedure. **A**) Preparation of the left internal mammary artery. **B**) Pericardial incision. **C**) Preparation of the left anterior descending artery. **D**) Preparing for the anastomosis. **E**) Anastomosis. **F**) Postoperative wound

Following induction of anaesthesia, the patient was put in a supine position with a 30° right lateral tilt. Using bipolar scissors, a small anterolateral thoracotomy was performed in the left fifth intercostal space. A Thoralift retractor was used to elevate the fifth rib for improved visualisation to facilitate harvesting the LIMA under direct vision. The LIMA was identified, and skeletonised dissection was carefully carried out without electrocoagulation. Side branches were freed and clipped precisely at a safe distance along the artery. The LIMA was prepared from its proximal origin to a standardised length of up to 15 cm. It was then prepared (Fig. 1.A) and dilated with papaverine solution; a good diameter and blood flow were confirmed. Following complete mobilization, the LIMA was temporarily clipped at its distal end.

After heparinisation, the pericardium was opened longitudinally (Fig. 1.B). The LAD was identified and the middle segment was exposed (Fig. 1.C). Stabilisation was achieved through correct positioning of the subxiphoid myocardial muscle stabiliser (Fehling Instruments GmbH & Co KG, Karlstein a. M., Germany). We proceeded with proximal slinging, and a stable circulation was confirmed.

A bulldog clamp was temporarily applied to the LIMA’s proximal end to prevent blood loss. The LIMA was transected, and a 4 mm incision was made at its distal end, with holding sutures placed to prepare for anastomosis (Fig. 1.D). A longitudinal 4 mm incision was made in the LAD. To achieve temporary occlusion, proximal throttling of the LAD was initiated with a snare suture at the site of anastomosis. The anastomosis of the LIMA was performed in an end-to-side fashion using a single continuous Prolene 8 − 0 suture (Fig. 1.E). The occlusion was subsequently released and perfusion of the LIMA was established. Flow measurement showed sufficient flow.

Heparinisation was reversed with 100% protamine, and vigilant haemostasis was carried out, keeping intraoperative blood loss minimal at less than 150 ml. To further minimise blood loss, a cell saver device was employed. A partial pericardial closure was performed, closing the remaining section by covering the distal segment of the LIMA with epicardial fat. This method was implemented to protect the LIMA during left lung inflation. A single chest drain was positioned in the left pleural cavity.

The thoracotomy was closed using the standard layer technique (Fig. 1.F). Following extubation on the first postoperative day, the chest drain was removed on the second postoperative day after accumulating a total output of 800 ml of serous fluid. The patient was transferred to the rehabilitation hospital on the fifth day. (Supplementary Video 1).

## Discussion

The primary goal of the case described was to perform the MIDCAB procedure without electrocoagulation, which is otherwise routinely used to achieve haemostasis at anastomotic sites. The concerns regarding the possible adverse effects on the patient’s cochlear implant [5], however, prompted a modified approach that allowed us to explore the feasibility of MIDCAB without relying on this common adjunct.

In a conventional CABG procedure in a multimorbid patient, increased bleeding and hence a greater need for electrocoagulation are anticipated. Our decision is consistent with emerging literature cautioning against the use of electrosurgical instruments in patients with cochlear implants in order to prevent potential adverse effects that have not yet been fully elucidated [5, 7]. In addition, studies have suggested that minimising electrocoagulation may be beneficial in preserving vascular integrity and reducing the risk of complications [8, 9].

In this particular case, the omission of electrocoagulation did not compromise the surgical outcome. The patient experienced no haemostatic issues pre- or postoperatively, no excessive bleeding, and reported no complaints at the 1-year postoperative follow-up. This suggests that, in selected cases, MIDCAB can be successfully performed without electrocoagulation.

Hybrid coronary revascularisation (HCR) represents a pivotal shift in cardiac surgery in that it combines the established therapeutic approaches of MIDCAB for LAD lesions and PCI with stenting for non-LAD lesions to achieve functionally complete revascularisation. As a compelling alternative to the conventional surgical strategies, it recognises that an ideal revascularisation strategy should be minimally invasive whilst prioritising expeditious patient recovery and minimising morbidity to enhance long-term survival rates.

Since its institution as a safe and reproducible procedure with good early and midterm results in 1996 [1], MIDCAB has achieved patency rates that are equivalent to those of conventional CABG [10, 11], and has been established as superior in the treatment of multivessel disease in the SYNTAX trial [12]. The advances in minimally invasive revascularisation strategies were, for the first time, incorporated into the 2018 ESC/EACTS guidelines on myocardial revascularisation [13].

Angelini et al. pioneered HCR [14]. Since then, various studies on the use, safety and efficacy of HCR have been published, concluding that it is a feasible and safe treatment option in MVCAD [15], giving patients an advantage in the long-term follow-up over those who undergo CABG [16]. The introduction of drug-eluting stents and the demonstration of their superior clinical outcomes and lower rates of restenosis have further advocated the hybrid approach and enhanced its feasibility [17].

Given the long-term durability of the LIMA-to-LAD graft accomplished through MIDCAB, HCR is currently deemed a viable alternative to other conventional treatment options [18]. However, hybrid approaches still account for less than 2% of all coronary revascularisations [19]. Despite its potential benefits, HCR faces limited adoption due to numerous logistic and practical concerns: challenges in coordinating procedure timing and sequence, difficulties in coordinating surgical and interventional groups, and concerns about bleeding with the use of aggressive anticoagulation [20].

Van Praet et al. outlined the recent inclusion criteria for HCR based on the currently available evidence: “just over 60 years of age; mainly stable, CAD favourable anatomy; intermediate risk and SYNTAX scores; and preserved or mildly impaired left ventricular ejection fraction” [21]. Contraindications include severe COPD (FVC < 60%), severe pulmonary hypertension, actively ischaemic patients, prior left thoracotomy and a history of pericarditis [20].

Due to the unique modification described in this case report, it is important to acknowledge the single-patient nature of this case report and, most importantly, the absence of a comparative group. Further research is warranted to establish guidelines for the judicious use of electrocoagulation in MIDCAB procedures, taking into account patient-specific factors and procedural nuances.

## Electronic supplementary material

Below is the link to the electronic supplementary material.

Supplementary Material 1

## Data Availability

No datasets were generated or analysed during the current study.

## Abbreviations

ASA: American Society of Anesthesiology
BCIG: British Cochlear Implant Group
CABG: Coronary artery bypass grafting
CAD: Coronary artery disease
COPD: Chronic obstructive pulmonary disease
CPB: Cardiopulmonary bypass
D1: First diagonal branch of LAD
ESC/EACTS: European Society of Cardiology – European Association for Cardio-Thoracic Surgery
HCR: Hybrid coronary revascularisation
LAD: Left anterior descending coronary artery
LCA: Left coronary artery
LIMA: Left internal mammary artery
MIDCAB: Minimally invasive direct coronary artery bypass
MRSA: Methicillin-resistant Staphylococcus aureus
MVCAD: Multivessel coronary artery disease
NYHA: New York Heart Association
PCI: Percutaneous coronary intervention
RCA: Right coronary artery
RCX: Left circumflex artery
SYNTAX Trial: Synergy Between Percutaneous Coronary Intervention with TAXUS and Cardiac Surgery
